# Supplementary material for: Self-assembling T7 phage syringes with modular genomes for targeted delivery of penicillin against β-lactam-resistant Escherichia coli
Source: BMC Biotechnol. 2025 Jul 1;25:63. doi: 10.1186/s12896-025-01003-2 (PMC12220360; doi:10.1186/s12896-025-01003-2)
Supplement: Supplementary file 1 — Supplementary Material 1 [file 12896_2025_1003_MOESM1_ESM.pdf]

Supplementary Information: Tables and Figures

Self-assembling T7 phage syringes with modular genomes deliver penicillin to neutralize β-lactam-resistant *Escherichia coli*

Hyunjin Shim[1,*]

Author Information

Affiliations

[1]Department of Biology, California State University, Fresno, 5241 N Maple Ave, Fresno, CA 93740, USA

*Corresponding author: Hyunjin Shim (shim@csufresno.edu)

Orcid links

Hyunjin Shim orcid=0000-0002-7052-0971

Figure S1. TEM images of T7 phages and phage syringes.

Imaging of each rebooted phage sample was performed using Talos F200C G2 fixed on a carbon-coated 400-mesh grid with negative staining. (A) Rebooted standard T7 phages. (B) Rebooted T7 phage syringes with all proteins (PSAP). (C) Rebooted T7 phage syringes with 1 μL penicillin (PS1P). In each image, examples of phage structures have been indicated with an arrow and text. T7 has a capsid that is icosahedral (twenty triangular faces) with an inner diameter of 55 nm and a short tail attached to the capsid in the form of podoviruses.

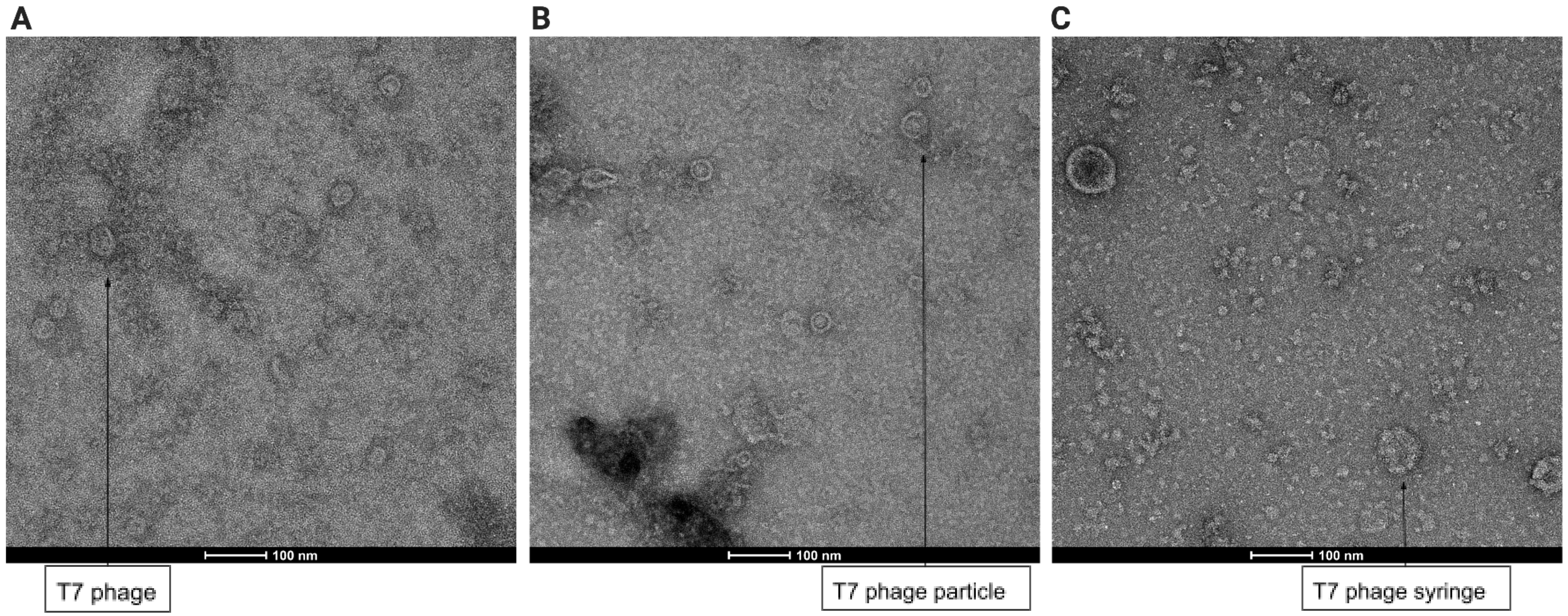

Table S1: Locus details of proteins expressed for phage syringes.

Locus tag	Product	Protein ID	Protein sequence
T7p41	HNH endonuclease	NP_041994.1	MEDCIEWTGGVNSKGYGRKWVNGKLVTPHRHIYEETYGPVPTGIVVMHICDNPRCYNIKHLTLGTPKDNSEDMVTKGRQAKGEELSKKLTESDVLAIRSSTLSHRSLGELYGVSQSTITRILQRKTWRHI
T7p42	head-tail adaptor	NP_041995.1	MAEKRTGLAEDGAKSVYERLKNDRAPYETRAQNCAQYTIPSLFPKDSDNASTDYQTPWQAVGARGLNNLASKLMLALFPMQTWMRLTISEYEAKQLLSDPDGLAKVDEGLSMVERIIMNYIESNSYRVTLFEALKQLVVAGNVLLYLPEPEGSNYNPMKLYRLSSYVVQRDAFGNVLQMVTRDQIAFGALPEDIRKAVEGQGGEKKADETIDVYTHIYLDEDSGEYLRYEEVEGMEVQGSDGTYPKEACPYIPIRMVRLDGESYGRSYIEEYLGDLRSLENLQEAIVKMSMISSKVIGLVNPAGITQPRRLTKAQTGDFVTGRPEDISFLQLEKQADFTVAKAVSDAIEARLSFAFMLNSAVQRTGERVTAEEIRYVASELEDTLGGVYSILSQELQLPLVRVLLKQLQATQQIPELPKEAVEPTISTGLEAIGRGQDLDKLERCVTAWAALAPMRDDPDINLAMIKLRIANAIGIDTSGILLTEEQKQQKMAQQSMQMGMDNGAAALAQGMAAQATASPEAMAAAADSVGLQPGI
T7p43	head assembly	NP_041996.1	MAESNADVYASFGVNSAVMSGGSVEEHEQNMLALDVAARDGDDAIELASDEVETERDLYDNSDPFGQEDDEGRIQVRIGDGSEPTDVDTGEEGVEGTEGSEEFTPLGETPEELVAASEQLGEHEEGFQEMINIAAERGMSVETIEAIQREYEENEELSAESYAKLAEIGYTKAFIDSYIRGQEALVEQYVNSVIEYAGGRERFDALYNHLETHNPEAAQSLDNALTNRDLATVKAIINLAGESRAKAFGRKPTRSVTNRAIPAKPQATKREGFADRSEMIKAMSDPRYRTDANYRRQVEQKVIDSNF
T7p44	major head protein	NP_041997.1	MASMTGGQQMGTNQGKGVVAAGDKLALFLKVFGGEVLTAFARTSVTTSRHMVRSISSGKSAQFPVLGRTQAAYLAPGENLDDKRKDIKHTEKVITIDGLLTADVLIYDIEDAMNHYDVRSEYTSQLGESLAMAADGAVLAEIAGLCNVESKYNENIEGLGTATVIETTQNKAALTDQVALGKEIIAALTKARAALTKNYVPAADRVFYCDPDSYSAILAALMPNAANYAALIDPEKGSIRNVMGFEVVEVPHLTAGGAGTAREGTTGQKHVFPANKGEGNVKVAKDNVIGLFMHRSAVGTVKLRDLALERARRANFQADQIIAKYAMGHGGLRPEAAGAVVFQSGVMLGVASTVAASPEEASVTSTEETLTPAQEAARTRAANKARKEAELAAATAEQ
T7p45	major head protein	NP_041998.1	MASMTGGQQMGTNQGKGVVAAGDKLALFLKVFGGEVLTAFARTSVTTSRHMVRSISSGKSAQFPVLGRTQAAYLAPGENLDDKRKDIKHTEKVITIDGLLTADVLIYDIEDAMNHYDVRSEYTSQLGESLAMAADGAVLAEIAGLCNVESKYNENIEGLGTATVIETTQNKAALTDQVALGKEIIAALTKARAALTKNYVPAADRVFYCDPDSYSAILAALMPNAANYAALIDPEKGSIRNVMGFEVVEVPHLTAGGAGTAREGTTGQKHVFPANKGEGNVKVAKDNVIGLFMHRSAVGTVKLRDLALERARRANFQADQIIAKYAMGHGGLRPEAAGAVVFKVE
T7p46	tail protein	NP_041999.1	MRSYDMNVETAAELSAVNDILASIGEPPVSTLEGDANADAANARRILNKINRQIQSRGWT

			FNIEEGITLLPDVYSNLIVYSDDYLSLMSTSGQSIYVNRGGYVYDRTSQSDRFDSGITVNI IRLRDYDEMPECFRYWIVTKASRQFNNRFFGAPEVEGVLQEEEDEARRLCMEYEMDY GGYNMLDGDAFTSGLLTR
T7p47	Tail protein	NP_042000.1	MALISQSIKNLKGGISQQPDILRYPDQGSRQVNGWSSETEGLQKRPPLVFLNTLGDNGA LGQAPYIHLINRDEHEQYYAVFTGSGIRVFDLSGNEKQVRYPNGSNYIKTANPRNDLRM VTVADYTFIVNRNVVAQKNTKSVNLPNYNPNQDGLINVRGGQYGRELIVHINGKDVAKY KIPDGSQPEHVNNTDAQWLAEELAKQMRTNLSDWTVNVGQGFIHVTAPSGQQIDSFTT KDGYADQLINPVTHYAQSFSKLPPNAPNGYMVKIVGDASKSADQYYVRYDAERKVWTE TLGWNTEDQVLWETMPHALVRAADGNFDFKWLEWSPKSCGDVDTNPWPSFVGSSIN DVFFFRNRLGFLSGENIILSRTAKYFNFYPASIANLSDDDPIDVAVSTNRIAILKYAVPFSEE LLIWSDEAQFVLTASGTLTSKSVELNLTTQFDVQDRARPFGIGRNVYFASPRSSFTSIHR YYAVQDVSSVKNAEDITSHVPNYIPNGVFSICGSGTENFCSVLSHGDPSKIFMYKFLYLN EELRQQSWSHWDFGENVQVLACQSISSDMYVILRNEFNTFLARISFTKNAIDLQGEPYR AFMDMKIRYTIPSGTYNDDTFTTSIHIPTIYGANFGRGKITVLEPDGKITVFEQPTAGWNS DPWLRLSGNLEGRMVYIGFNINFVYEFSKFLIKQTADDGSTSTEDIGRLQLRRAWVNYE NSGTFDIYVENQSSNWKYTMAGARLGSNTLRAGRLNLGTGQYRFPVVGNAKFNTVYIL SDETTPLNIIGCGWEGNYLRRSSGI
T7p48	internal virion protein	NP_042001.1	MMTIRPTKSTDFEVFTPAHHDILEAKAAGIEPSFPDASECVTLSLYGFPLAIGGNCGDQC WFVTSDQVWRLSGKAKRKFRKLIMEYRDKMLEKYDTLWNYVWVGNTSHIRFLKTIGAV FHEEYTRDGQFQLFTITKGG
T7p49	internal virion protein	NP_042002.1	MCWAAAIPIAISGAQAISGQNAQAKMIAAQTAAGRRQAMEIMRQTNIQNADLSLQARSK LEEASAELTSQNMQKVQAIGSIRAAIGESMLEGSSMDRIKRVTEGQFIREANMVTENYR RDYQAIFAQQLGGTQSAASQIDEIYKSEQKQKSKLQMVLDPLAIMGSSAASAYASGAFD SKSTTKAPIVAAKGTKTGR
T7p50	internal virion protein	NP_042003.1	MSKIESALQAAQPGLSRLRGGAGGMGYRAATTQAEQPRSSLLDTIGRFAKAGADMYTA KEQRARDLADERSNEIIRKLTPEQRREALNNGTLLYQDDPYAMEALRVKTGRNAAYLVD DDVMQKIKEGVFRTREEMEEYRHSRLQEGAKVYAEQFGIDPEDVDYQRGFNGDITERN ISLYGAHDNFLSQQAQKGAIMNSRVELNGVLQDPDMLRRPDSADFFEKYIDNGLVTGAI PSDAQATQLISQAFSDASSRAGGADFLMRVGDKKVTLNGATTTYRELIGEEQWNALMV TAQRSQFETDAKLNEQYRLKINSALNQEDPRTAWEMLQGIKAELDKVQPDEQMTPQRE WLISAQEQVQNQMNAWTKAQAKALDDSMKSMNKLDVIDKQFQKRINGEWVSTDFKDM PVNENTGEFKHSDMVNYANKKLAEIDSMDIPDGAKDAMKLKYLQADSKDGAFRTAIGT MVTDAGQEWSAAVINGKLPERTPAMDALRRIRNADPQLIAALYPDQAELFLTMDMMDK QGIDPQVILDADRLTVKRSKEQRFEDDKAFESALNASKAPEIARMPASLRESARKIYDSV KYRSGNESMAMEQMTKFLKESTYTFTGDDVDGDTVGVIPKNMMQVNSDPKSWEQGR DILEEARKGIIASNPWITNKQLTMYSQGDSIYLMDTTGQVRVRYDKELLSKVWSENQKKL EEKAREKALADVNKRAPIVAATKAREAAAKRVREKRKQTPKFIYGRKE
T7p51	internal virion protein with	NP_042004.1	MDKYDKNVPSDYDGLFQKAADANGVSYDLLRKVAWTESRFVPTAKSKTGPLGMMQFT

	endolysin domain		KATAKALGLRVTDGPDDDRLNPELAINAAAKQLAGLVGKFDGDELKAALAYNQGEGRLGNPQLEAYSKGDFASISEEGRNYMRNLLDVAKSPMAGQLETFGGITPKGKGIPAEVGLAGIGHKQKVTQELPESTSFDVKGIEQEATAKPFAKDFWETHGETLDEYNSRSTFFGFKNAAEAELSNSVAGMAFRAGRLDNGFDVFKDTITPTRWNSHIWTPEELEKIRTEVKNPAYINVVTGGSPENLDDLIKLANENFENDSRAAEAGLGAKLSAGIIGAGVDPLSYVPMVGVTGKGFKLINKALVVGAESAALNVASEGLRTSVAGGDADYAGAALGGFVFGAGMSAISDAVAAGLKRSKPEAEFDNEFIGPMMRLEARETARNANSADLSRMNTENMKFEGEHNGVPYEDLPTERGAVVLHDGSVLSASNPINPKTLKEFSEVDPEKAARGIKLAGFTEIGLKTLGSDDADIRRVAIDLVRSPTGMQSGASGKFGATASDIHERLHGTDQRTYNDLYKAMSDAMKDPEFSTGGAKMSREETRYTIYRRAALAIERPELQKALTPSERIVMDIIKRHFDTKRELMENPAIFGNTKAVSIFPESRHKGTYVPHVYDRHAKALMIQRYGAEGLQEGIARSWMNSYVSRPEVKARVDEMLKELHGVKEVTPEMVEKYAMDKAYGISHSDQFTNSSIIEENIEGLVGIENNSFLEARNLFDSDLSITMPDGQQFSVNDLRDFDMFRIMPAYDRRVNGDIAIMGSTGKTTKELKDEILALKAKAEGDGKKTGEVHALMDTVKILTGRARRNQDTVWETSLRAINDLGFFAKNAYMGAQNITEIAGMIVTGNVRALGHGIPILRDTLYKSKPVSAKELKELHASLFGKEVDQLIRPKRADIVQRLREATDTGPAVANIVGTLKYSTQELAARSPWTKLLNGTTNYLLDAARQGMLGDVISATLTGKTTRWEKEGFLRGASVTPEQMAGIKSLIKEHMVRGEDGKFTVKDKQAFSMDPRAMDLWRLADKVADEAMLRPHKVSLQDSHAFGALGKMVMQFKSFTIKSLNSKFLRTFYDGYKNNRAIDAALSIITSMGLAGGFYAMAAHVKAYALPKEKRKEYLERALDPTMIAHAALSRSSQLGAPLAMVDLVGGVLGFESSKMARSTILPKDTVKERDPNKPYTSREVMGAMGSNLLEQMPSAGFVANVGATLMNAAGVVNSPNKATEQDFMTGLMNSTKELVPNDPLTQQLVLKIYEANGVNLRERRK
T7p52	tail fiber protein	NP_042005.1	MANVIKTVLTYQLDGSNRDFNIPFEYLARKFVVVTLIGVDRKVLTINTDYRFATRTTISLTKAWGPADGYTTIELRRVTSTTDRLVDFTDGSILRAYDLNVAQIQTMHVAEEARDLTTDTIGVNNDGHLDARGRRIVNLANAVDDRDAVPFGQLKTMNQNSWQARNEALQFRNEAETFRNQAEGFKNESSTNATNTKQWRDETKGFRDEAKRFKNTAGQYATSAGNSASAAHQSEVNAENSATASANSAHLAEQQADRAEREADKLENYNGLAGAIDKVDGTNVYWKGNIHANGRLYMTTNGFDCGQYQQFFGGVTNRYSVMEWGDENGWLMYVQRREWTTAIGGNIQLVVNGQIITQGGAMTGQLKLQNGHVLQLESASDKAHYILSKDGNRNNWYIGRGSDNNNDCTFHSYVHGTTLTLKQDYAVVNKHFHVGQAVVATDGNIQGTKWGGKWLDAYLRDSFVAKSKAWTQVWSGSAGGGVSVTVSQDLRFRNIWIKCANNSWNFFRTGPDGIYFIASDGGWLRFQIHSNGLGFKNIADSRSVPNAIMVENE
T7p53	holin	NP_042006.1	MLSLDFNNELIKAAPIVGTGVADVSARLFFGLSLNEWFYVAAIAYTVVQIGAKVVDKMIDWKKANKE
T7p54	terminase small subunit	NP_042007.1	MEKDKSLITFLEMLDTAMAQRMLADLSDHERRSPQLYNAINKLLDRHKFQIGKLQPDVHILGGLAGALEEYKEKVGDNGLTDDDIYTLQ
T7p55	Rz-like spanin	NP_042008.1	MLEFLRKLIPWVLAGMLFGLGWHLGSDSMDAKWKQEVHNEYVKRVEAAKSTQRAIDAVSAKYQEDLAALEGSTDRIISDLRSDNKRLRVRVKTTGTSDGQCGFEPDGRAELDDRDAKRILAVTQKGDAWIRALQDTIRELQRK

T7p56		NP_042009.1	MSTLRELRLRRALKEQSMRYLLSIKKTLPRWKGALIGLFLICVATISGCASESKLPEPPMVSVDSSLMVEPNLTTEMLNVFSQ
T7p57	terminase large subunit	NP_042010.1	MSTQSNRNALVVAQLKGDFVAFLFVLWKALNLPVPTKCQIDMAKVLANGDNKKFILQAFRGIGKSFITCAFVVWSLWRDPQLKILIVSASKERADANSIFIKNIIDLLPFLSELKPRPGQRDSVISFDVGPANPDHSPSVKSVGITGQLTGSRADIIIADDVEIPSNSATMGAREKLWTLVQEFAALLKPLPSSRVIYLGTPQTEMTLYKELEDNRGYTTIIWPALYPRTREENLYYSQRLAPMLRAEYDENPEALAGTPTDPVRFDRDDLRERELEYGKAGFTLQFMLNPNLSDAEKYPLRLRDAIVAALDLEKAPMHYQWLPNRQNIIEDLPNVGLKGDDLHTYHDCSNNSGQYQQKILVIDPSGRGKDETGYAVLYTLNGYIYLMEAGGFRDGYSDKTLELLAKKAKQWGVQTVVYESNFGDGMFGKVFSPILLKHHNCAMEEIRARGMKEMRICDTLEPVMQTHRLVIRDEVIRADYQSARDVDGKHDVKYSLFYQMTRITREKGALAHDDRLDALALGIEYLRESMQLDSVKVEGEVLADFLEEHMMRPTVAATHIIEMSVGGVDVYSEDDEGYGTSFIEW
T7p58	hypothetical protein	NP_042011.1	MGTQPLSGLLCTQGHVKRTSITHSVLLLCYALSTMRTLRHLLGLQQTQCALTVMTCASVSWNTVRLALRYSSCLTLTLVMPRSTR
T7p59	hypothetical protein	NP_042012.1	MATPIRPLSYSLRRQSNGESRRLSTRVTSVTVCSVRYSVLSFLNTTTVRWKRFVPVV
T7p60	hypothetical protein	NP_042013.1	MFRLLLNLLRHRVTYRFLVVLCAALGYASLTGDLSSLESVVCSILTCSD

Table S2: Time-serial measurements of normalized OD600 readings of antimicrobial susceptibility testing using a spectrophotometer.

Time-point	PSAP	PSAP	PSAP	PSSP	PSSP	PSSP	PS1P	PS1P	PS1P	PS2P	PS2P	PS2P	PS10P	PS10P	PS10P	PC	PC	PC	NC	NC	NC	Pen10ul	Pen10ul	Pen10ul	Pen2ul	Pen2ul	Pen2ul	Pen1ul	Pen1ul	Pen1ul
**1**	1.00	1.00	1.00	1.00	1.00	1.00	1.00	1.00	1.00	1.00	1.00	1.00	1.00	1.00	1.00	1.00	1.00	1.00	1.00	1.00	1.00	1.00	1.00	1.00	1.00	1.00	1.00	1.00	1.00	1.00
**2**	0.77	0.13	0.11	0.96	1.00	1.00	0.34	1.00	0.65	0.27	0.97	1.00	0.32	1.00	0.46	0.04	0.14	0.01	0.77	1.03	0.93	1.04	0.91	0.20	0.67	0.81	0.17	0.86	0.23	1.00
**3**	1.00	0.08	0.06	0.75	0.99	0.96	0.18	0.95	0.62	0.13	0.97	1.00	0.39	1.00	1.00	0.03	0.10	0.01	0.63	1.04	0.96	1.01	0.92	0.08	0.00	0.62	-0.17	0.81	0.14	0.28
**4**	0.14	0.10	0.06	0.65	0.87	0.13	0.12	0.31	0.01	0.09	0.25	0.95	0.21	0.97	0.15	0.06	0.08	0.01	0.88	1.02	0.97	0.95	0.97	0.06	0.33	0.67	0.00	0.65	0.14	0.20
**5**	0.05	0.04	0.03	0.52	0.59	0.00	0.10	0.24	0.00	0.07	0.34	0.07	0.21	1.00	0.00	0.03	0.07	0.01	0.45	0.83	0.90	0.89	0.93	0.03	0.67	0.65	0.17	0.72	0.09	0.16
**6**	0.01	0.05	0.05	0.41	0.48	0.00	0.12	0.17	0.01	0.05	0.14	0.01	0.21	0.43	0.00	0.01	0.06	0.00	0.36	0.71	0.80	0.74	1.00	0.03	0.33	0.30	0.17	0.64	0.05	0.16
**7**	0.01	0.00	0.00	0.31	0.10	0.00	0.01	0.14	0.01	0.02	0.09	0.00	0.19	0.25	0.01	0.00	0.05	0.00	0.34	0.62	0.88	0.87	0.93	0.02	0.33	0.29	0.50	0.34	0.14	0.11
**8**	0.00	-0.03	-0.03	0.24	0.06	-0.03	0.02	0.13	0.01	0.01	0.07	0.01	0.10	0.19	0.00	0.01	0.04	0.00	0.33	0.61	0.82	0.61	0.73	0.05	0.67	0.18	0.33	0.32	0.00	0.08
**9**	0.02	-0.04	-0.03	0.21	0.02	-0.04	0.00	0.07	0.01	0.02	0.06	0.01	0.15	0.19	0.00	0.01	0.03	0.00	0.24	0.45	0.57	0.50	0.64	0.00	0.33	0.15	0.33	0.38	-0.05	0.14
**10**	0.00	-0.04	-0.03	0.16	-0.02	-0.04	0.00	0.09	0.01	0.00	0.07	0.00	0.09	0.16	0.01	0.01	0.04	0.00	0.21	0.37	0.48	0.58	0.71	0.02	0.33	0.13	0.33	0.17	0.09	0.06
**11**	0.00	NA	NA	0.13	-0.01	NA	0.00	0.04	0.00	0.00	0.04	0.01	0.06	0.10	0.01	0.00	0.02	0.00	0.29	0.45	0.41	0.35	0.61	0.00	0.00	0.06	0.17	0.06	0.09	0.00
**12**	0.00	NA	NA	0.10	NA	NA	0.00	0.03	0.00	0.00	0.03	0.01	0.03	0.11	0.00	0.00	0.01	0.00	0.15	0.43	0.55	0.39	0.38	0.00	0.33	0.01	0.00	0.04	0.05	0.00

Table S3: Quality control of protein during the process of phage rebooting for minimum inhibitory concentration testing.

Protein quality control of samples after cell-free protein expression with A280 and A260 readings from a spectrophotometer.

**Protein quality control after cell-free protein expression**				
**Sample Name**	**Concentration**	**Units**	**A280**	**A260/A280**
**Phage syringes with 1 µL penicillin (PS1P)**	573.663	mg/mL	80.32	2.11
**Phage syringes with 1 µL penicillin diluted in 9 µL PBS solution (PS1P_0.1)**	10.966	mg/mL	1.54	2.30
**Phage syringes with 1 µL penicillin diluted in 99 µL PBS solution (PS1P_0.01)**	4.584	mg/mL	0.64	2.21

Table S4: Time-serial measurements of normalized OD600 readings of antimicrobial susceptibility testing using a spectrophotometer.

PS1P_A and PS1P_B are the first biological replicate and the second replicate of phage syringes rebooted with 1 μL penicillin G, respectively. PS1P_A_mean and PS1P_A_sd show the average and the standard deviation of the three technical replicates of the first biological replicate (PS1P_A). PS1P_B_mean and PS1P_B_sd show the average and the standard deviation of the three technical replicates of the second biological replicate (PS1P_B).

OD600	PS1P_A	PS1P_A	PS1P_A	PS1P_B	PS1P_B	PS1P_B	PS1P_A_mean	PS1P_A_sd	PS1P_B_mean	PS1P_B_sd
**0**	1.00	1.00	1.00	1.00	1.00	1.00	**1.000**	**0.000**	**1.000**	**0.000**
**20**	0.34	1.00	0.65	1.06	1.00	0.89	**0.664**	**0.333**	**0.984**	**1.000**
**40**	0.18	0.95	0.62	0.28	1.00	0.98	**0.582**	**0.388**	**0.753**	**0.961**
**60**	0.12	0.31	0.01	0.28	0.40	0.93	**0.148**	**0.155**	**0.538**	**0.912**
**80**	0.10	0.24	0.00	0.06	0.60	0.78	**0.115**	**0.121**	**0.480**	**0.741**
**100**	0.12	0.17	0.01	0.22	0.60	0.78	**0.098**	**0.079**	**0.532**	**0.685**
**120**	0.01	0.14	0.01	0.28	0.60	0.76	**0.053**	**0.076**	**0.546**	**0.707**
**140**	0.02	0.13	0.01	0.28	0.75	0.58	**0.051**	**0.065**	**0.536**	**0.688**
**160**	0.00	0.07	0.01	0.03	0.60	0.51	**0.026**	**0.039**	**0.381**	**0.594**
**180**	0.00	0.09	0.01	0.03	0.50	0.51	**0.032**	**0.049**	**0.347**	**0.448**
**200**	0.00	0.04	0.00	0.00	0.45	0.40	**0.015**	**0.022**	**0.283**	**0.451**
**220**	0.00	0.03	0.00	-0.03	0.25	0.36	**0.014**	**0.017**	**0.191**	**0.355**

Table S5: Final measurements of raw OD600 readings of minimum inhibitory concentration testing using a spectrophotometer.

The last time point (tfinal) shows OD600 readings after 24 hours of incubation at 37℃ since the time of dosage.

**Normalized OD600**	**PS1P_1**	**PS1P_2**	**PS1P_3**	**PS1P_0.1_1**	**PS1P_0.1_2**	**PS1P_0.1_3**	**PS1P_0.01_1**	**PS1P_0.01_2**	**PS1P_0.01_3**	**PS1P_0.001_1**	**PS1P_0.001_2**	**PS1P_0.001_3**
**tfinal**	0.01	0.07	0.13	0.01	0.03	0.06	0.11	0.07	0.03	0.63	0.6	0.14
